# Supplementary material for: Limited carry-over effects of socioemotional manipulations on subsequent unrelated memory tasks
Source: PLoS One. 2024 Oct 31;19(10):e0309193. doi: 10.1371/journal.pone.0309193 (PMC11527296; doi:10.1371/journal.pone.0309193)
Supplement: S1 Table — (DOCX) [file pone.0309193.s002.docx]

| **Supplementary Table 1.** Education level as a function of Experiment and Condition | | | | | | | |
| --- | --- | --- | --- | --- | --- | --- | --- |
|  | *Experiment 1 (Neutral Images)* | | | | | | |
|  | Less than 8 years of formal education | Grade School  (8th grade) | Some high school | High school diploma | Some college | College degree | Professional degree |
| Control | 0 | 0.4 | 0.7 | 12.2 | 40.5 | 33 | 12.5 |
| Encoding-Music | 0 | 0 | 0.7 | 20.1 | 33.1 | 35.6 | 10.6 |
| Encoding-Self | 0 | 0 | 1.1 | 9.9 | 31.3 | 42 | 15.3 |
| Retrieval-Autobio | 0 | 0 | 0.7 | 13.6 | 33.1 | 38.7 | 13.9 |
| Retrieval-Music | 0 | 0 | 0.4 | 16.8 | 30.4 | 41.6 | 10.8 |
| Retrieval-Self | 0 | 0 | 1.2 | 11.2 | 30.4 | 45.4 | 11.9 |
|  | *Experiment 2 (Emotional Images)* | | | | | | |
|  | Less than 8 years of formal education | Grade School (8th grade) | Some high school | High school diploma | Some college | College degree | Professional degree |
| Control | 0 | 0 | 0 | 22.1 | 39.7 | 30.9 | 7.4 |
| Encoding-Music | 0 | 0 | 1.6 | 12.7 | 38.1 | 33.3 | 14.3 |
| Encoding-Self | 0 | 0 | 1.4 | 22.5 | 31 | 35.2 | 9.9 |
| Retrieval-Autobio | 0 | 0 | 0 | 15.4 | 49.2 | 27.7 | 7.7 |
| Retrieval-Music | 0 | 0 | 0 | 11.6 | 50.7 | 23.2 | 14.5 |
| Retrieval-Self | 0 | 0 | 1.4 | 16.4 | 35.6 | 38.4 | 8.2 |
| *Note*. Values represent percentage of group reporting each level of education. | | | | | | | |
