# Supplementary material for: Limited carry-over effects of socioemotional manipulations on subsequent unrelated memory tasks
Source: PLoS One. 2024 Oct 31;19(10):e0309193. doi: 10.1371/journal.pone.0309193 (PMC11527296; doi:10.1371/journal.pone.0309193)
Supplement: S2 Table — (DOCX) [file pone.0309193.s003.docx]

| **Supplementary Table 2.** Race and ethnicity as a function of Experiment and Condition | | | | | | | | |
| --- | --- | --- | --- | --- | --- | --- | --- | --- |
|  | *Experiment 1 (Neutral Images)* | | | | | | | |
|  | Hispanic | Not Hispanic | American Indian or Alaskan Native | Asian | Black | Native Hawaiian or other Pacific Islander | White | Other |
| Control | 6.5 | 93.5 | 0.0 | 13.2 | 1.5 | 0.0 | 77.9 | 7.4 |
| Encoding-Music | 7.0 | 93.0 | 0.0 | 14.3 | 6.3 | 0.0 | 79.4 | 0.0 |
| Encoding-Self | 5.3 | 94.7 | 0.0 | 14.1 | 5.6 | 0.0 | 80.3 | 0.0 |
| Retrieval-Autobio | 7.0 | 93.0 | 0.0 | 16.9 | 6.2 | 0.0 | 75.4 | 1.5 |
| Retrieval-Music | 7.2 | 92.8 | 0.0 | 7.2 | 4.3 | 0.0 | 85.5 | 2.9 |
| Retrieval-Self | 8.5 | 91.5 | 0.0 | 6.8 | 1.4 | 0.0 | 89.0 | 2.7 |
|  | *Experiment 2 (Emotional Images)* | | | | | | | |
|  | Hispanic | Not Hispanic | American Indian or Alaskan Native | Asian | Black | Native Hawaiian or other Pacific Islander | White | Other |
| Control | 7.4 | 92.6 | 0.0 | 6.5 | 5.7 | 0.0 | 84.6 | 2.9 |
| Encoding-Music | 4.8 | 95.2 | 0.7 | 6.0 | 6.3 | 0.0 | 84.2 | 2.8 |
| Encoding-Self | 7.0 | 93.0 | 0.4 | 8.4 | 5.0 | 0.4 | 84.4 | 1.5 |
| Retrieval-Autobio | 3.1 | 96.9 | 1.0 | 6.3 | 5.2 | 0.0 | 85.4 | 2.1 |
| Retrieval-Music | 2.9 | 97.1 | 1.2 | 6.8 | 5.2 | 0.0 | 82.8 | 4 |
| Retrieval-Self | 8.2 | 91.8 | 0.8 | 9.6 | 6.5 | 0.4 | 79.6 | 3.1 |
| *Note*. Values represent percentage of group who endorsed each race or ethnicity. | | | | | | | | |
