# Supplementary material for: Limited carry-over effects of socioemotional manipulations on subsequent unrelated memory tasks
Source: PLoS One. 2024 Oct 31;19(10):e0309193. doi: 10.1371/journal.pone.0309193 (PMC11527296; doi:10.1371/journal.pone.0309193)
Supplement: S1 File — Experiment 1 COVID timing (pre- v. post- shut down orders) Attention (Test of Everyday Attention, TEA) Community Integration (Community Integration Questionnaire, CIQ) Working Memory (n-back: 2-back v. 0-back performance) Sleep (Pittsburg Sleep Quality Index, PSQI) Experiment 2 Attention (Test of Everyday Attention; TEA) Community Integration (Community Integration Questionnaire, CIQ) Working Memory (n-back: 2-back v. 0-back performance) Crystallized Knowledge (Shipley Vocabulary Test) Sleep (Pittsburg Sleep Quality Index, PSQI) Depression (Geriatric Depression Scale, GDS, and Beck’s Depression Inventory, BDI) Loneliness (UCLA Loneliness Scale. (DOCX) [file pone.0309193.s004.docx]

Although the lack of a significant benefit might argue that socioemotional manipulations do not support better memory, it is also possible that they may be particularly beneficial for certain participants (e.g., those who are less able to rely on traditional learning strategies) or in certain circumstances (e.g., when instructions are presented at a particular timepoint during the task). Therefore, exploratory follow-up analyses were conducted to examine individual differences. Significant changes to manipulation effects are reported in the main text, but all other statistics are reported below. Bolded values are significant at *p*<.05.

Experiment 1

| *COVID timing* | | |
| --- | --- | --- |
|  | **Age** | ***F*(1,1609)= 7.57, *p*=.006, η^2^_p_=.005** |
|  | Manipulation timing | *F*(1,1609)= .28, *p*=.75, η^2^_p_<.001 |
|  | COVID | *F*(1,1609)= .74, *p*=.39, η^2^_p_<.001 |
|  | Age-by-Manipulation | *F*(1,1609)= 1.75, *p*=.18, η^2^_p_=.002 |
|  | **Age-by-COVID** | ***F*(1,1609)= 33.25, *p*<.001, η^2^_p_=.02** |
|  | COVID-by-Manipulation | *F*(1,1609)= 1.98, *p*=.14, η^2^_p_=.001 |
|  | Age-by-COVID-by-Manipulation | *F*(1,1609)= .88, *p*=.41, η^2^_p_=.001 |
| *Test of Everyday Attention (TEA)* | | |
|  | **Age** | ***F*(1,1068)= 41.61, *p*<.001, η^2^_p_=.04** |
|  | Manipulation timing | *F*(1,1068)= .24, *p*=.62, η^2^_p_<.001 |
|  | **TEA** | ***F*(1,1068)= 49.35, *p*<.001, η^2^_p_=.04** |
|  | Age-by-Manipulation | *F*(1,1068)= .61, *p*=.37, η^2^_p_=.001 |
|  | **Age-by-TEA** | ***F*(1,1068)= 17.00, *p*<.001, η^2^_p_=.02** |
|  | TEA-by-Manipulation | *F*(1,1068)= .10, *p*=.75, η^2^_p_<.001 |
|  | Age-by-TEA-by-Manipulation | *F*(1,1068)= .07, *p*=.79, η^2^_p_<.001 |
| *Community Integration Questionnaire (CIQ)* | | |
|  | **Age** | ***F*(1,724)= 21.61, *p*<.001, η^2^_p_=.03** |
|  | Manipulation timing | *F*(1,724)= .09, *p*=.91, η^2^_p_<.001 |
|  | CIQ | *F*(1,724)= 1.94, *p*=.16, η^2^_p_=.003 |
|  | Age-by-Manipulation | *F*(1,724)= 1.55, *p*=.21, η^2^_p_=.004 |
|  | **Age-by-CIQ** | ***F*(1,724)= 7.17, *p*=.008, η^2^_p_=.01** |
|  | CIQ-by-Manipulation | *F*(1,724)= .07, *p*=.93, η^2^_p_<.001 |
|  | Age-by-CIQ-by-Manipulation | *F*(1,724)= 1.07, *p*=.34, η^2^_p_=.003 |
| *N-Back* | | |
|  | **Age** | ***F*(1,1609)= 65.63, *p*<.001, η^2^_p_=.04** |
|  | Manipulation timing | *F*(1,1609)= 2.78, *p*=.06, η^2^_p_=.003 |
|  | **Nback** | ***F*(1,1609)= 12.98, *p*<.001, η^2^_p_=.008** |
|  | Age-by-Manipulation | *F*(1,1609)= 2.02, *p*=.13, η^2^_p_=.003 |
|  | **Age-by-Nback** | ***F*(1,1609)= 7.52, *p*=.006, η^2^_p_=.005** |
|  | Nback-by-Manipulation | *F*(1,1609)= 2.80, *p*=.06, η^2^_p_=.003 |
|  | Age-by-Nback-by-Manipulation | *F*(1,1609)= 2.02, *p*=.13, η^2^_p_=.003 |
| *Sleep* | | |
|  | **Age** | ***F*(1,1304)= 62.90, *p*<.001, η^2^_p_=.05** |
|  | **Manipulation timing** | ***F*(1,1304)= 4.01, *p*=.02, η^2^_p_=.006** |
|  | Sleep | *F*(1,1304)= .35, *p*=.56, η^2^_p_<.001 |
|  | **Age-by-Manipulation** | ***F*(1,1304)= 3.94, *p*=.02, η^2^_p_=.006** |
|  | **Age-by-Sleep** | ***F*(1,1304)= 4.48, *p*=.03, η^2^_p_=.003** |
|  | Sleep-by-Manipulation | *F*(1,1304)= 1.85, *p*=.16 |
|  | Age-by-Sleep-by-Manipulation | *F*(1,1304)= .24, *p*=.79, η^2^_p_<.001 |

Experiment 2

| *Test of Everyday Attention (TEA)* | | |
| --- | --- | --- |
|  | Age | *F*(1,267)= 0.01, *p*=.93, η^2^_p_<.001 |
|  | Manipulation timing | *F*(1,267)= 0.29, *p*=.59, η^2^_p_=.001 |
|  | **TEA** | ***F*(1,267)= 6.52, *p*=.01, η^2^_p_=.024** |
|  | **Valence** | ***F*(1,267)= 5.39, *p*=.02, η^2^_p_=.02** |
|  | Age-by-Manipulation | *F*(1,267)= 2.54, *p*=.11, η^2^_p_=.009 |
|  | Age-by-TEA | *F*(1,267)= .40, *p*=.53, η^2^_p_=.001 |
|  | TEA-by-Manipulation | *F*(1,267)= 1.07, *p*=.30, η^2^_p_=.004 |
|  | Age-by-Valence | *F*(1,267)= 1.64, *p*=.20 η^2^_p_=.006 |
|  | Manipulation-by-Valence | *F*(1,267)= 0.04, *p*=.85, η^2^_p_<.001 |
|  | TEA-by-Valence | *F*(1,267)= 2.70, *p*=.10, η^2^_p_=.01 |
|  | Age-by-TEA-by-Manipulation | *F*(1,267)= 2.98, *p*=.09, η^2^_p_=.01 |
|  | Age-by-TEA-by-Valence | *F*(1,267)= .13, *p*=.72, η^2^_p_<.001 |
|  | Age-by-Manipulation-by-Valence | *F*(1,267)= 0.13, *p*=.72, η^2^_p_=.001 |
|  | Manipulation-by-TEA-by-Valence | *F*(1,267)= 1.94, *p*=.17, η^2^_p_=.007 |
|  | Age-by-Manipulation-by-TEA-by-Valence | *F*(1,267)= .02, *p*=.89, η^2^_p_<.001 |
| *Community Integration Questionnaire (CIQ)* | | |
|  | Age | *F*(1,396)= 0.02, *p*=.89, η^2^_p_<.001 |
|  | Manipulation timing | *F*(1,396)= 0.28, *p*=.76, η^2^_p_=.001 |
|  | **CIQ** | ***F*(1,396)= 4.29, *p*=.04, η^2^_p_=.01** |
|  | **Valence** | ***F*(1,396)= 9.24, *p*=.003, η^2^_p_=.02** |
|  | Age-by-Manipulation | *F*(1,396)= 0.67, *p*=.52, η^2^_p_=.003 |
|  | Age-by-CIQ | *F*(1,396)= 1.21, *p*=.27, η^2^_p_=.003 |
|  | CIQ-by-Manipulation | *F*(1,396)= .76, *p*=.47, η^2^_p_=.004 |
|  | **Age-by-Valence** | ***F*(1,396)= 5.43, *p*=.02, η^2^_p_=.01** |
|  | Manipulation-by-Valence | *F*(2,396)= 0.27, *p*=.76, η^2^_p_=.001 |
|  | CIQ-by-Valence | *F*(1,396)= 0.05, *p*=.82, η^2^_p_<.001 |
|  | Age-by-CIQ-by-Manipulation | *F*(1,396)= 1.11, *p*=.33, η^2^_p_=.006 |
|  | Age-by-CIQ-by-Valence | *F*(1,396)= 3.41, *p*=.07, η^2^_p_=.009 |
|  | Age-by-Manipulation-by-Valence | *F*(2,396)= 0.33, *p*=.97, η^2^_p_<.001 |
|  | Manipulation-by-CIQ-by-Valence | *F*(1,396)= 0.65, *p*=.52, η^2^_p_=.003 |
|  | Age-by-Manipulation-by-CIQ-by-Valence | *F*(1,396)= 1.87, *p*=.16, η^2^_p_=.009 |
| *Working Memory (Nback)* | | |
|  | Age | *F*(1,397)< .001, *p*>.99, η^2^_p_<.001 |
|  | Manipulation timing | *F*(1,397)= .97, *p*=.38, η^2^_p_=.005 |
|  | Nback | *F*(1,397)= 3.15, *p*=.08, η^2^_p_=.008 |
|  | **Valence** | ***F*(1,397)= 9.18, *p*=.003, η^2^_p_=.02** |
|  | Age-by-Manipulation | *F*(1,397)= .58, *p*=.56, η^2^_p_=.003 |
|  | Age-by-Nback | *F*(1,397)= 1.26, *p*=.26, η^2^_p_=.003 |
|  | Nback-by-Manipulation | *F*(1,397)= 1.02, *p*=.36, η^2^_p_=.005 |
|  | Age-by-Valence | *F*(1,397)= 2.31, *p*=.13, η^2^_p_=.006 |
|  | Manipulation-by-Valence | *F*(1,397)= .13, *p*=.88, η^2^_p_=.001 |
|  | Nback-by-Valence | *F*(1,397)= .003, *p*=.96, η^2^_p_<.001 |
|  | Age-by-Nback-by-Manipulation | *F*(1,397)= .60, *p*=.55, η^2^_p_=.003 |
|  | Age-by-Nback-by-Valence | *F*(1,397)= .005, *p*=.94, η^2^_p_<.001 |
|  | Age-by-Manipulation-by-Valence | *F*(1,397)= .13, *p*=.88, η^2^_p_=.001 |
|  | Manipulation-by-Nback-by-Valence | *F*(1,397)= .26, *p*=.77, η^2^_p_=.001 |
|  | Age-by-Manipulation-by-Nback-by-Valence | *F*(1,397)= 1.92, *p*=.15, η^2^_p_=.01 |
| *Shipley* | | |
|  | **Age** | ***F*(1,397)= 4.31, *p*=.04, η^2^_p_=.01** |
|  | Manipulation timing | *F*(1,397)= 0.73, *p*=.48, η^2^_p_=.004 |
|  | **Shipley** | ***F*(1,397)= 26.43, *p*<.001, η^2^_p_=.06** |
|  | Valence | *F*(1,397)= 1.65, *p*=.20, η^2^_p_=.004 |
|  | Age-by-Manipulation | *F*(1,397)= 0.54, *p*=.58, η^2^_p_=.003 |
|  | Age-by-Shipley | *F*(1,397)= 0.49, *p*=.48, η^2^_p_=.001 |
|  | Shipley-by-Manipulation | *F*(1,397)= 0.28, *p*=.75, η^2^_p_=.001 |
|  | **Age-by-Valence** | ***F*(1,397)= 4.73, *p*=.03, η^2^_p_=.01** |
|  | Manipulation-by-Valence | *F*(1,397)= 0.63, *p*=.53, η^2^_p_=.003 |
|  | Shipley-by-Valence | *F*(1,397)= 2.15, *p*=.14, η^2^_p_=.005 |
|  | Age-by-Shipley-by-Manipulation | *F*(1,397)= 0.64, *p*=.53, η^2^_p_=.003 |
|  | **Age-by-Shipley-by-Valence** | ***F*(1,397)= 4.41, *p*=.04, η^2^_p_=.01** |
|  | Age-by-Manipulation-by-Valence | *F*(1,397)= 0.50, *p*=.61, η^2^_p_=.002 |
|  | Manipulation-by-Shipley-by-Valence | *F*(1,397)= 1.09, *p*=.34, η^2^_p_=.005 |
|  | Age-by-Manipulation-by-Shipley-by-Valence | *F*(1,397)= 1.15, *p*=.32, η^2^_p_=.006 |
| Pittsburg Sleep Quality Index (PSQI) | | |
|  | Age | *F*(1,318)= 0.07, *p*=.80, η^2^_p_<.001 |
|  | Manipulation timing | *F*(1,318)= 0.32, *p*=.73, η^2^_p_=.002 |
|  | PSQI | *F*(1,318)= 1.41, *p*=.24, η^2^_p_=.004 |
|  | **Valence** | ***F*(1,318)= 11.53, *p*=.001, η^2^_p_=.04** |
|  | Age-by-Manipulation | *F*(1,318)= 0.77, *p*=.46, η^2^_p_=.005 |
|  | Age-by-PSQI | *F*(1,318)= 2.91, *p*=.09, η^2^_p_=.009 |
|  | PSQI-by-Manipulation | *F*(1,318)= 1.49, *p*=.23, η^2^_p_=.009 |
|  | Age-by-Valence | *F*(1,318)= 2.75, *p*=.10, η^2^_p_=.009 |
|  | Manipulation-by-Valence | *F*(1,318)= 0.08, *p*=.93, η^2^_p_<.001 |
|  | PSQI-by-Valence | *F*(1,318)= 0.89, *p*=.35, η^2^_p_=.003 |
|  | Age-by-PSQI-by-Manipulation | *F*(1,318)= 1.36, *p*=.26, η^2^_p_=.008 |
|  | Age-by-PSQI-by-Valence | *F*(1,318)= 2.14, *p*=.15, η^2^_p_=.007 |
|  | Age-by-Manipulation-by-Valence | *F*(1,318)= 0.06, *p*=.95, η^2^_p_<.001 |
|  | Manipulation-by-PSQI-by-Valence | *F*(1,318)= 0.93, *p*=.40, η^2^_p_=.006 |
|  | Age-by-Manipulation-by-PSQI-by-Valence | *F*(1,318)= 1.66, *p*=.19, η^2^_p_=.01 |
| Geriatric Depression Scale (GDS) | | |
|  | Age | *F*(1,396)= 0.06, *p*=.81, η^2^_p_<.001 |
|  | Manipulation timing | *F*(1,396)= 0.67, *p*=.51, η^2^_p_=.003 |
|  | GDS | *F*(1,396)= 0.53, *p*=.47, η^2^_p_=.001 |
|  | **Valence** | ***F*(1,396)= 11.59, *p*=.001, η^2^_p_=.03** |
|  | Age-by-Manipulation | *F*(1,396)= 0.47, *p*=.63, η^2^_p_=.002 |
|  | Age-by-GDS | *F*(1,396)= 0.02, *p*=.90, η^2^_p_<.001 |
|  | GDS-by-Manipulation | *F*(1,396)= 0.91, *p*=.40, η^2^_p_=.005 |
|  | Age-by-Valence | *F*(1,396)= 2.99, *p*=.09, η^2^_p_=.007 |
|  | Manipulation-by-Valence | *F*(1,396)= 0.08, *p*=.93, η^2^_p_<.001 |
|  | GDS-by-Valence | *F*(1,396)= 2.15, *p*=.14, η^2^_p_=.005 |
|  | Age-by-GDS-by-Manipulation | *F*(1,396)= 1.82, *p*=.16, η^2^_p_=.009 |
|  | Age-by-GDS-by-Valence | *F*(1,396)= 1.03, *p*=.31, η^2^_p_=.003 |
|  | Age-by-Manipulation-by-Valence | *F*(1,396)= 0.09, *p*=.91, η^2^_p_<.001 |
|  | **Manipulation-by-GDS-by-Valence** | ***F*(1,396)= 3.71, *p*=.03, η^2^_p_=.02** |
|  | Age-by-Manipulation-by-GDS-by-Valence | *F*(1,396)= 0.32, *p*=.73, η^2^_p_=.002 |
| *Beck’s Depression Inventory (BDI)* | | |
|  | Age | *F*(1,396)= 0.09, *p*=.77, η^2^_p_<.001 |
|  | Manipulation timing | *F*(1,396)= 0.48, *p*=.62, η^2^_p_=.002 |
|  | BDI | *F*(1,396)= 0.29, *p*=.59, η^2^_p_=.001 |
|  | **Valence** | ***F*(1,396)= 11.67, *p*=.001, η^2^_p_=.03** |
|  | Age-by-Manipulation | *F*(1,396)= 0.15, *p*=.86, η^2^_p_=.001 |
|  | Age-by-BDI | *F*(1,396)= 0.80, *p*=.37, η^2^_p_=.002 |
|  | BDI-by-Manipulation | *F*(1,396)= 0.61, *p*=.54, η^2^_p_=.003 |
|  | Age-by-Valence | *F*(1,396)= 2.37, *p*=.12, η^2^_p_=.006 |
|  | Manipulation-by-Valence | *F*(1,396)= 0.13, *p*=.88, η^2^_p_=.001 |
|  | BDI-by-Valence | *F*(1,396)= 1.37, *p*=.24, η^2^_p_=.003 |
|  | Age-by-BDI-by-Manipulation | *F*(1,396)= 1.52, *p*=.22, η^2^_p_=.008 |
|  | Age-by-BDI-by-Valence | *F*(1,396)= 0.09, *p*=.77, η^2^_p_<.001 |
|  | Age-by-Manipulation-by-Valence | *F*(1,396)= 0.11, *p*=.89, η^2^_p_=.001 |
|  | **Manipulation-by-BDI-by-Valence** | ***F(1,396)= 2.92, p=.06, η^2^_p_=.02*** |
|  | Age-by-Manipulation-by-BDI-by-Valence | *F*(1,396)= 0.46, *p*=.63, η^2^_p_=.002 |
| *UCLA Loneliness Scale (UCLA)* | | |
|  | Age | *F*(1,395)= 0.05, *p*=.83, η^2^_p_<.001 |
|  | Manipulation timing | *F*(1,395)= 1.10, *p*=.33, η^2^_p_=.006 |
|  | UCLA | *F*(1,395)= 0.002, *p*=.97, η^2^_p_<.001 |
|  | Valence | ***F*(1,395)= 10.96, *p*=.001, η^2^_p_=.03** |
|  | Age-by-Manipulation | *F*(1,395)= 0.92, *p*=.40, η^2^_p_=.005 |
|  | Age-by-UCLA | *F*(1,395)< 0.001, *p*=.99, η^2^_p_<.001 |
|  | UCLA-by-Manipulation | *F*(1,395)= 2.39, *p*=.09, η^2^_p_=.01 |
|  | Age-by-Valence | ***F*(1,395)= 4.18, *p*=.04, η^2^_p_=.01** |
|  | Manipulation-by-Valence | *F*(1,395)= 0.17, *p*=.85, η^2^_p_=.001 |
|  | UCLA-by-Valence | *F*(1,395)= 1.13, *p*=.29, η^2^_p_=.003 |
|  | Age-by-UCLA-by-Manipulation | *F*(1,395)= 1.82, *p*=.16, η^2^_p_=.009 |
|  | Age-by-UCLA-by-Valence | ***F*(1,395)= 4.18, *p*=.04, η^2^_p_=.01** |
|  | Age-by-Manipulation-by-Valence | *F*(1,395)= 0.17, *p*=.84, η^2^_p_=.001 |
|  | Manipulation-by-UCLA-by-Valence | *F*(1,395)= 1.17, *p*=.31, η^2^_p_=.006 |
|  | Age-by-Manipulation-by-UCLA-by-Valence | ***F*(1,395)= 3.18, *p*=.04, η^2^_p_=.02** |
