# Supplementary material for: Limited carry-over effects of socioemotional manipulations on subsequent unrelated memory tasks
Source: PLoS One. 2024 Oct 31;19(10):e0309193. doi: 10.1371/journal.pone.0309193 (PMC11527296; doi:10.1371/journal.pone.0309193)
Supplement: S2 File — (DOCX) [file pone.0309193.s005.docx]

| *Autobiographical memory manipulation* | | |
| --- | --- | --- |
|  | Valence | *F*(1,129)= 1.11, *p*=.30, η^2^_p_=.009 |
|  | Age | *F*(1,129)= 0.11, *p*=.74, η^2^_p_=.001 |
|  | Manipulation timing | *F*(1,129)= 0.26, *p*=.61, η^2^_p_=.002 |
|  | Age-by-timing | *F*(1,129)= 2.29, *p*=.13, η^2^_p_=.02 |
|  | Valence-by-age | *F*(1,129)= 0.82, *p*=.37, η^2^_p_=.006 |
|  | Valence-by-timing | *F*(1,129)= 0.32, *p*=.58, η^2^_p_=.002 |
|  | Valence-by-age-by-timing | *F*(1,129)= 0.07, *p*=.79, η^2^_p_=.001 |
| *Music manipulation* | | |
|  | **Valence** | ***F*(1,194)= 5.34, *p*=.02, η^2^_p_=.03** |
|  | Age | *F*(1,194)= 0.02, *p*=.88, η^2^_p_<.001 |
|  | Manipulation timing | *F*(2,194)= 1.03, *p*=.36, η^2^_p_=.01 |
|  | Age-by-timing | *F*(2,194)= 0.70, *p*=.50, η^2^_p_=.007 |
|  | Valence-by-age | *F*(1,194)= 1.17, *p*=.28, η^2^_p_=.006 |
|  | Valence-by-timing | *F*(2,194)= 0.76, *p*=.47, η^2^_p_=.008 |
|  | Valence-by-age-by-timing | *F*(2,194)= 0.04, *p*=.96, η^2^_p_<.001 |
| *Self-reference manipulation* | | |
|  | **Valence** | ***F*(1,206)= 12.84, *p*<.001, η^2^_p_=.06** |
|  | Age | *F*(1,206)= 0.20, *p*=.66, η^2^_p_=.001 |
|  | Manipulation timing | *F*(2,206)= 0.07, *p*=.93, η^2^_p_=.001 |
|  | Age-by-timing | *F*(2,206)= 0.48, *p*=.62, η^2^_p_=.005 |
|  | Valence-by-age | *F*(1,206)= 3.34, *p*=.07, η^2^_p_=.02 |
|  | Valence-by-timing | *F*(2,206)= 0.46, *p*=.63, η^2^_p_=.004 |
|  | Valence-by-age-by-timing | *F*(2,206)= 0.35, *p*=.71, η^2^_p_=.003 |
