# Supplementary material for: Limited carry-over effects of socioemotional manipulations on subsequent unrelated memory tasks
Source: PLoS One. 2024 Oct 31;19(10):e0309193. doi: 10.1371/journal.pone.0309193 (PMC11527296; doi:10.1371/journal.pone.0309193)
Supplement: S3 File — (DOCX) [file pone.0309193.s006.docx]

*Vividness* was calculated by averaging together participants’ vividness ratings for *hits only*. In each Experiment, we conducted a Factorial ANCOVA with *manipulation* (pre-encoding, pre-retrieval, and none/control) as a between-subjects factor and *age* (treated as a continuous variable) as a covariate of interest. In Experiment 2, valence (i.e, positive v. negative) was included as an additional within-subject factor. We were interested in the effect of the manipulation, as well as the interaction of manipulation with age.

In Experiment 1, vividness was not influenced by age (*F*(1,1615)= .02, *p*=.90, η^2^_p_<.001) or the age-by-manipulation interaction (*F*(1,1615)= .89, *p*=.41, η^2^_p_=.001), but there was a trending effect of manipulation (*F*(1,1615)= 2.89, *p*=.06, η^2^_p_=.006), where participants in the control condition rated memories as more vivid (*M*=3.95, *SE*=.05) than those who had a memory manipulation prior to encoding (*M*=3.82, *SE*=.06).

Participants in Experiment 2 provided higher vividness ratings for positive (*M*=4.14, *SE*=.04) relative to negative items (*M*=4.00, *SE*=.04; *F*(1,403)=60.10, *p*<.001, η^2^_p_=.13), but this did not differ as a function of age (*F*(1,403)=.29, *p*=.59, η^2^_p_=.001), manipulation (*F*(2,403)=1.90, *p*=.15, η^2^_p_=.009), or the age-by-manipulation interaction (*F*(2,403)=.38, *p*=.68, η^2^_p_=.002). There was not a significant main effect of age (*F*(1,403)=.02, *p*=.90, η^2^_p_<.001) or manipulation (*F*(2,403)=.08, *p*=.92, η^2^_p_<.001), or an interaction of these factors (*F*(2,403)=.75, *p*=.47, η^2^_p_=.004).

When comparing Experiments 1 and 2, vividness ratings did not differ as a function of age (*F*(1,2018)= .03, *p*=.87, η^2^_p_<.001) or manipulation (*F*(1,2018)= .45, *p*=.64, η^2^_p_<.001), but there was a significant effect of Experiment (*F*(1,2018)= 12.25, *p*<.001, η^2^_p_=.006), where ratings were higher for Experiment 2 (*Emotional images; M*=4.07, *SE*=.05) compared to Experiment 1 (*Neutral images; M*=3.89, *SE*=.02). There were no significant interactions in this analysis (age-by-manipulation: *F*(1,2018)= .05, *p*=.95, η^2^_p_<.001; manipulation-by-experiment: *F*(1,2018)= .83, *p*=.44, η^2^_p_=.001; age-by-experiment: *F*(1,2018)= .001, *p*=.97, η^2^_p_<.001; age-by-manipulation-by-experiment: *F*(1,2018)= 1.2*9*, *p*=.28, η^2^_p_=.001).
