# Supplementary material for: Limited carry-over effects of socioemotional manipulations on subsequent unrelated memory tasks
Source: PLoS One. 2024 Oct 31;19(10):e0309193. doi: 10.1371/journal.pone.0309193 (PMC11527296; doi:10.1371/journal.pone.0309193)
Supplement: S4 File — (DOCX) [file pone.0309193.s007.docx]

A supplementary analysis examined the differential effect of manipulation on neutral (Ex 1) and emotional (Ex 2) images *within the same group of participants*. 351 participants from Experiment 1 also completed the Experiment 2 survey (ages 19-77, *M*= 42.44, *SD*=18.24, 50% female, 61% with a college degree or more, 77% white and 93% not Hispanic). These participants were not included in any of the reported analyses for Experiment 2.

Of these participants, 45 were in the *Control* condition in Experiment 1 and a *Memory Manipulation* condition in Experiment 2 (ages 22-72, *M*= 44.4, *SD*=17.36, 51% female, 67% with a college degree or more, 80% white and 93% not Hispanic), and 53 were in a *Memory Manipulation* condition in Experiment 1 and the *Control* condition in Experiment 2 (ages 21-69, *M*= 42.23, *SD*=16.48, 49% female, 66% with a college degree or more, 74% white and 94% not Hispanic). These two groups did not differ as a function of age (*F*(1,96)=.40, *p*=.53), sex (χ^2^(1)= .04, *p*=.84), race (χ^2^(4)= 1.23, *p*=.87), or ethnicity (χ^2^(1)= .04, *p*=.84). However, participants who were in the Memory Manipulation condition first had significantly higher levels of education (χ^2^(4)= 10.69, *p*=.03).

Because the sample size in each bin was too small to conduct an analysis of manipulation timing (i.e., pre-encoding v. pre-retrieval), this analysis collapsed across all manipulation conditions relative to control. This analysis included time (Experiment 1 v. Experiment 2) and group (Control first v. Manipulation first) as between-subject factors and age as a continuous covariate.

As in other analyses, age was associated with increased accuracy in this analysis (*F*(1,94)=7.97, *p*=.006, η^2^_p_=.08), qualified by significant time-by-age (*F*(1,94)=4.34, *p*=.04, η^2^_p_=.04) and time-by-age-by-group (*F*(1,94)=9.18, *p*=.003, η^2^_p_=.09) interactions. Correlations were conducted to better understand these interactions. Age was associated with similar age-related increases for both Experiments in individuals who completed the Manipulation condition first (*r*=.27, *p*=.05 and *r*=.33, *p*=.02 for Ex 1 and Ex 2, respectively). However, for participants who completed the Control condition first, age was associated with greater accuracy in Experiment 1 (*r*=.37, *p*=.01) but not Experiment 2 (*r*=.06, *p*=.72). The main effect of time (*F*(1,94)=.26, *p*=.61, η^2^_p_=.003) and the time-by-group interaction (*F*(1,94)=.08, *p*=.79, η^2^_p_=.001) were not significant. Accuracy also did not differ across groups (*F*(1,94=1.10, *p*=.30, η^2^_p_=.01) and the effect of age did not differ across groups (*F*(1,94)=.14, *p*=.71, η^2^_p_=.002).

Vividness ratings did not differ as a function of age (*F*(1,94)=.15, *p*=.70, η^2^_p_=.002), group (*F*(1,94)=.78, *p*=.38, η^2^_p_<.001), or time (*F*(1,94)=2.55, *p*=.11, η^2^_p_=.03), and all interactions were not significant (age-by-group: *F*(1,94)=.15, *p*=.70, η^2^_p_=.002; time-by-group: *F*(1,94)=2.74, *p*=.10, η^2^_p_=.03; *F*(1,94)=.15, *p*=.70, η^2^_p_=.002; age-by-time: *F*(1,94)=.82, *p*=.37, η^2^_p_=.009; age-by-time-by-group: *F*(1,94)=.51, *p*=.48, η^2^_p_=.005).
