# Supplementary material for: Limited carry-over effects of socioemotional manipulations on subsequent unrelated memory tasks
Source: PLoS One. 2024 Oct 31;19(10):e0309193. doi: 10.1371/journal.pone.0309193 (PMC11527296; doi:10.1371/journal.pone.0309193)
Supplement: Supplementary file 7 [file pone.0309193.s001.docx]

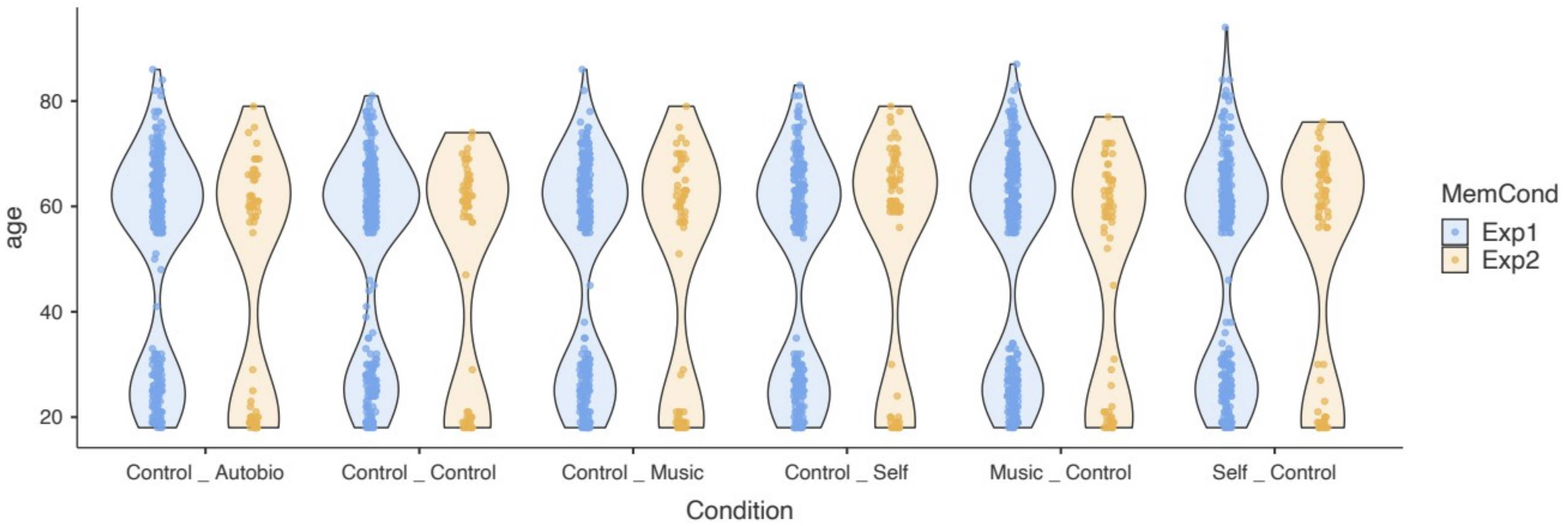

age
80
60
40
20
Control _ Autobio
Control _ Control
Control _ Music
Control _ Self
Music _ Control
Self _ Control
Condition
MemCond
Exp1
Exp2
