# Supplementary material for: Limited carry-over effects of socioemotional manipulations on subsequent unrelated memory tasks
Source: PLoS One. 2024 Oct 31;19(10):e0309193. doi: 10.1371/journal.pone.0309193 (PMC11527296; doi:10.1371/journal.pone.0309193)
Supplement: Attachment — Submitted filename: Response to Reviewers.docx [file pone.0309193.s008.docx]

July 16, 2024

Emily Chenette

Editor-in-Chief, *PLOS ONE*

Dear Dr. Chenette,

Thank you for reviewing our manuscript entitled, “Limited carry-over effects of socioemotional manipulations on subsequent unrelated memory tasks“. We sincerely appreciated the suggestions provided by the academic editor and 2 reviewers, as well as their positive response to the manuscript.

As stated in the cover letter, the revised manuscript has been edited to conform with PLOS ONE’s stye requirements. The editor also requested an edit to the Role of Funder statement, stating that: “The funders had no role in study design, data collection and analysis, decision to publish, or preparation of the manuscript.” We have addressed the reviewers’ comments below and have incorporated these changes into the manuscript (in red). We hope that you will now find this manuscript suitable for publication in *PLOS ONE.*

Sincerely,

Jaclyn Hennessey Ford

**Reviewer #1**

1. The reviewer requested a clearer connection between the introduction and the specific objectives of the study.

Response: The introduction has been updated to include more connections between the literature and the current research questions.

2. The reviewer noted that the introduction could be more direct in pointing out the specific gap in the literature that the study addresses. For example, they asked for an explicit statement as to how the current research goes beyond previous studies on socioemotional memory in the elderly.

Response: The current study is the first to consider the use of socioemotional tasks as a manipulation to establish an ideal encoding or retrieval mode for older adults. Other research has shown a benefit of socioemotional processing *during* memory tasks, but not prior to memory. This information has been highlighted in the introduction

3. The reviewer requested more information about the age distribution of participants.

Response: We have added violin plots of the age distribution as a function of condition and experiment.

4. The reviewer requested more information about the instructions that participants were given for the socioemotional manipulations.

Response: We have added the exact instructions provided to participants to the methods section of the manuscript.

5. The reviewer requested a figure or flowchart to illustrate the flow of the experiment

Response: We have updated Fig 1 to better reflect the flow of the experiment.

6. The reviewer requested additional tables and figures to visualize the data.

Response: The manuscript includes a table or figure for all reported findings, so we were not sure what other visualization the reviewer was hoping for. However, we would be happy to add an additional figure or table if the review has a specific request.

7. The reviewer requested a concise synthesis of the main findings of the exploratory analyses.

Response: The results section has been updated to include summaries of the findings in each paragraph.

8. The reviewer requested additional suggestions for future research

Response: Suggestions for future research have been added throughout the discussion section

9. The reviewer noted that the figures should be saved at a higher quality

Response: The figures have been saved at a higher quality

10. The reviewer noted a lack of recent (2023 and 2024) references

Response: We have added a number of new references to keep the manuscript up-to-date.

11. The reviewer requested an expanded version of the conclusions paragraph

Response: The conclusions paragraph now includes a more thorough discussion of the theoretical and methodological contributions of the study, as well as a review of future research.

**Reviewer #2**

1. The reviewer noted that the introduction heading is missing

Response: This heading has been added

2. The reviewer suggested focusing on four additional points to enhance the final conclusion paragraph, as it felt short and out of balance with the rest of the manuscript:
   a. First, they asked how the current findings might contribute to overarching theory.
   b. Second, they requested a discussion of the practical contributions of the study to practitioners in the field
   c. Third, the reviewer asked that the limitations, discussed elsewhere in the discussion, be mentioned in the conclusion.
   d. Finally, they request suggestions for future research

Response: The conclusions paragraph has been updated according to these suggestions.

3. The reviewer noted that the manuscript was missing references from the past two years

Response: We have added a number of new references to keep the manuscript up-to-date.

4. The reviewer requested additional focus on the theoretical contributions of the study at the end of the manuscript

Response: A description of the theoretical contributions has been added as part of the updated conclusions
